# Translational framework for implementation evaluation and research: a normalisation process theory coding manual for qualitative research and instrument development

**DOI:** 10.1186/s13012-022-01191-x

**Published:** 2022-02-22

**Authors:** Carl R. May, Bianca Albers, Mike Bracher, Tracy L. Finch, Anthony Gilbert, Melissa Girling, Kathryn Greenwood, Anne MacFarlane, Frances S. Mair, Christine M. May, Elizabeth Murray, Sebastian Potthoff, Tim Rapley

**Affiliations:** 1 Department of Health Services Research and Policy, London School of Hygiene and Tropical Medicine & NIHR North Thames ARC, London, UK; 2 Institute for Implementation Science in Healthcare, Zurich, Switzerland; 3 School of Health Sciences, University of Southampton, Southampton, UK; 4 Department of Nursing, Midwifery & Health, Northumbria University & NIHR ARC North East-North Cumbria, Newcastle, UK; 5 Royal National Orthopaedic Hospital, London & NIHR North Thames ARC, London, UK; 6 School of Psychology, University of Sussex, Brighton, UK; 7 School of Medicine and Health Research Institute, University of Limerick, Limerick, Ireland; 8 Institute of Health and Wellbeing, Glasgow University, Glasgow, UK; 9 Southampton, UK; 10 Research Department of Primary Care and Population Health, University College London & NIHR North Thames ARC, London, UK; 11 Department of Social Work, Education and Community Wellbeing, Northumbria University & NIHR ARC North East- North Cumbria, Newcastle, UK

## Abstract

**Background:**

Normalisation Process Theory (NPT) is frequently used to inform qualitative research that aims to explain and evaluate processes that shape late-stage translation of innovations in the organisation and delivery of healthcare. A coding manual for qualitative researchers using NPT will facilitate transparent data analysis processes and will also reduce the cognitive and practical burden on researchers.

**Objectives:**

(a) To simplify the theory for the user. (b) To describe the purposes, methods of development, and potential application of a coding manual that translates normalisation process theory (NPT) into an easily usable framework for qualitative analysis. (c) To present an NPT coding manual that is ready for use.

**Method:**

Qualitative content analysis of papers and chapters that developed normalisation process theory, selection and structuring of theory constructs, and testing constructs against interview data and published empirical studies using NPT.

**Results:**

A coding manual for NPT was developed. It consists of 12 primary NPT constructs and conforms to the Context-Mechanism-Outcome configuration of realist evaluation studies. Contexts are defined as settings in which implementation work is done, in which strategic intentions, adaptive execution, negotiating capability, and reframing organisational logics are enacted. Mechanisms are defined as the work that people do when they participate in implementation processes and include coherence-building, cognitive participation, collective action, and reflexive monitoring. Outcomes are defined as effects that make visible how things change as implementation processes proceed and include intervention mobilisation, normative restructuring, relational restructuring, and sustainment.

**Conclusion:**

The coding manual is ready to use and performs three important tasks. It consolidates several iterations of theory development, makes the application of NPT simpler for the user, and links NPT constructs to realist evaluation methods. The coding manual forms the core of a translational framework for implementation research and evaluation.

**Supplementary Information:**

The online version contains supplementary material available at 10.1186/s13012-022-01191-x.

Contribution to the literature

- Normalisation Process Theory is widely used to design complex interventions, and to understand the dynamics of implementation processes and their outcomes.
- Normalisation Process Theory has been developed through several iterations, and this paper consolidates these into a single, empirically grounded, translational framework for implementation and evaluation research.
- This paper describes the development of a Normalisation Process Theory coding manual for qualitative research and instrument design, and it presents the coding manual ready to use.
- The coding manual links the primary constructs of Normalisation Process Theory to the Context-Mechanism-Outcome framework of realist evaluation.

## Background

*Any researcher who wishes to become proficient at doing qualitative analysis must learn to code well and easily. The excellence of the research rests in large part on the excellence of the coding.*

Anselm Strauss [1]

The analysis and interpretation of qualitative data can make an important contribution to research on implementation processes and their outcomes when such data are interpreted through the lens of implementation theory. These data may be found in documents, interview transcripts, or observational fieldnotes. In broad terms, there are two approaches to integrating qualitative methods and implementation theory. First, by explaining phenomena of interest through procedures that identify and characterise empirical regularities or deviant cases in natural language data through processes of induction [1]. Second, by deriving explanations of relevant phenomena through using structured methods of data analysis that directly engage with existing conceptual frameworks, models, and theories [2–5]. These are not mutually exclusive ways of working, and they are often combined. In this paper, we focus on developing tools for the second approach, in which a more structured approach to qualitative data analysis [6] was formed into a coding manual that supports researchers using Normalisation Process Theory (NPT) [7–11] in studies of implementation processes.

NPT provides a set of conceptual tools that support understanding and evaluation of the adoption, implementation, and sustainment of socio-technical and organisational innovations. NPT takes as its starting point that implementation processes are formed when actors seek to translate their strategic intentions into ensembles of beliefs, behaviours, artefacts, and practices that create change in the everyday practices of others [8, 11]. The central questions that follow from the application of NPT are always, *what is the work that actors do to create change*? *How does this work get done*? And, *what are its effects*? Because NPT has its origins in research on the implementation of complex healthcare interventions, it does not see the intervention as a thing-in-itself, but rather as an assemblage or ensemble of beliefs, behaviours, artefacts, and practices that may play out differently over time and between settings [8]. It is supported by empirical studies using both qualitative and quantitative methods and by systematic reviews that have explored its value in different research domains [12–14].

Development of the coding manual was informed by the application of methods of qualitative content analysis described by Schreier [2]. This approach can be defined as ‘a research method for the subjective interpretation of the content of text-data through the systematic classification process of coding and identifying themes or patterns’ [3] and as ‘any qualitative data reduction and sense-making effort that takes a volume of qualitative material and attempts to identify core consistencies and meanings.’ [4]. As qualitative content analysis has become more widely used, so too have coding frameworks and manuals that define the ways that data are identified, categorised, and characterised within a study. In qualitative content analysis, researchers are encouraged to develop manuals that describe and explicate definition of the ‘rules’ for coding and categorising data [5]. The process of categorisation that follows from using a coding manual is useful because it enables researchers to manage the cognitive burden of searching for and handling multiple constructs and thus enables them to manage a greater cognitive burden of interpretation. Within research teams, coding manuals support the quality and rigour of coding by providing ‘rules’ that are employed by each team member and, in this way, can ensure the consistency of coding. Parsimony can be important too: more is not necessarily better in qualitative investigation and analysis. Reducing the number of codes to those that represent core constructs can be understood as what Adams et al. [6], in a different context, have called ‘subtractive transformation’.

A generalizable NPT coding manual is of value to researchers from a range of disciplines interested in the ways that implementation processes play out. It provides a consistent set of definitions of the core constructs of the theory, shows how they relate to each other, and enables researchers doing qualitative content analysis together to work within a common frame of analysis (for example, in qualitative evidence syntheses, or in team-based qualitative analysis of interview or observational data). In the future, as software for computational hermeneutics [15] becomes more widely available and practically workable, a coding manual could also be integrated into the development of topic modelling instruments and algorithms.

Despite their value to researchers, the process of creating rigorous and robust coding manuals for individual studies is rarely described, and generalizable coding manuals are rare. In this paper, we start to fill this gap. We describe the purposes, methods of development, and application of a generalizable coding manual that translates NPT into a more easily usable framework for qualitative analysis.

## Methods

NPT has developed over time through contact with empirical studies and evidence syntheses, and this has led to different iterations of the theory. These have been formed through publications that have served three purposes. First, there is a set of papers aimed explicitly at *theory-building* in which core constructs of NPT have been developed and their implications explored [7–9, 16, 17]. Second, there is a set of papers aimed explicitly at *theory-translation* in which those core constructs have been clarified and refined through methodological research leading to the development of toolkits [18, 19] and survey instruments [20–22]. Finally, there is a set of papers that contribute to *theory-elaboration* through the development of new constructs during empirical studies and systematic reviews. These explain additional aspects of implementation processes [23–25].

Translating a set of theoretical constructs into a theory-informed coding manual for qualitative data analysis involves a series of tasks that are, in themselves, a form of qualitative analysis. Qualitative research focuses on the identification, characterisation, and interpretation of empirical regularities or deviant cases in natural language data. The process described here developed organically and opportunistically through these different tasks, as they were conducted, and through discussion amongst authors of this paper. The work of defining key constructs of the theory, assembling these into a framework, and then transforming them into a workable coding manual, was informed by the qualitative content analysis procedures described by Schreier [2].

1. *Concept identification*. The result of the iterative development of NPT is a body of constructs representing the mechanisms that motivate and shape implementation processes, the outcomes of these processes, and the contexts in which their users make them workable and integrate them into practice. These core constructs of NPT were distributed over papers that developed the theory [7–11, 16, 17, 23–25] and in others that developed the means and methods of its application [18–22]. In June 2020, CRM assembled these constructs in a taxonomy of statements (*n*=149). They identified, characterised, and explained observable features of the collective action and collaborative work of implementation (the taxonomy of NPT statements is presented in the online supplementary material),
2. *De-duplication and disambiguation*. The taxonomy of 149 statements assembled in selection and structuring work included multiple duplicates, along with ambiguous and overlapping descriptions of constructs. CRM identified duplicate, ambiguous, and overlapping constructs. These were then either disambiguated or eliminated. After this work was competed, 38 discrete constructs were retained to make up a ‘first pass’ coding manual (the ‘first pass’ coding manual is presented in the online supplementary material).
3. *Piloting*. The ‘first pass’ manual was piloted. CRM used it to code two papers selected from an earlier NPT systematic review. These were comprehensively coded and checked by all the authors of this paper, who critically commented on codes and coding decisions. The same coding manual was then applied to two sets of interview data collected in other studies that were informed by NPT. First, AG coded transcripts of interviews (*n*=55 with managers, practitioners, and patients) conducted for an evaluation of the accelerated implementation of remote clinician-patient interaction in a tertiary orthopaedic centre during the COVID-19 pandemic. Second, KG coded transcripts of interviews (*n*=22, with community mental health professionals) conducted for the process evaluation of the EYE-2 Trial (an engagement intervention for first episodes of psychosis employed in early intervention in the community) [26].
4. *Further disambiguation*. Pilot work demonstrated that the main elements of the ‘first pass’ coding manual were workable in practice. The piloting exercise revealed that the first pass coding manual was hard to use because it was over-complex and because it micro-managed the process of interpretation. This defeated attempts at nuanced interpretation. Additional work to disambiguate constructs and eliminate overlapping or redundant ones was therefore undertaken as we worked through steps 5–8, below.
5. *Identification of context-related constructs*. Within the coding manual the *contexts* in which implementation work takes place remained invisible, although taking context into account had been an important element of theory development and elaboration over time [10, 11]. The contexts of implementation can be understood as *both* structures *and* processes [27]. To remedy the absence of constructs representing context, CRM returned to the taxonomy of 149 NPT constructs and the first pass coding manual and searched them for salient descriptors of context. Four of these were identified and were added to the manual.
6. *Further piloting*. The four constructs relating to implementation contexts were piloted ‘in use’ by CRM on a set of interview transcripts (*n*=36) collected in a study of professionals’ participation in the implementation of treatment escalation plans to manage care at the end of life in British hospitals [28]. It was found that these constructs characterised process contexts effectively.
7. *Presentation*. The structure of the coding manual was then presented and discussed in a series of international webinars in February–April 2021. Discussion with participants in those webinars assisted in clarifying the ways that NPT constructs fitted together and characterised actual processes and outcomes.
8. *Agreement*. Once the final structure of the coding manual was laid out, all authors then read and commented on it. This led to further ruthless editing and simplification of the coding manual.
9. *Post-submission*. Journal peer review is intended to improve papers for publication. In this case, it led to a clearer and more coherent presentation of the methods leading to the development of the coding manual and of the coding manual itself. An important outcome of this process was further simplification of the construct descriptors in Tables 1 and 2. These were also linked to their primary sources in the NPT literature.
10. *‘Living peer review’*. Between the initial submission of a manuscript to Implementation Science (2 September 2021) and the finalisation of the manuscript (December 30, 2021), the first draft of the coding manual was viewed or downloaded more than 1600 times from the preprint servers ResearchSquare.com and ResearchGate.net. This led to useful feedback from researchers who began to use the coding manual to do ‘real-world’ data analysis as soon as it became available but who did not have specific NPT expertise. As a result of this ‘living peer review’, further simplification of the descriptions of NPT constructs was undertaken by CRM.

**Table 1:** NPT coding manual part A: primary constructs—contexts, mechanisms, and outcomes

CMO domain	NPT construct	Description and example
Implementation contexts: Contexts are patterns of social relations and structures that unfold over time and across settings. They make up the implementation environment.	**Strategic intentions** [11]	**Description:** How do contexts shape the formulation and planning of interventions and their components? [11]. **Example:** ‘The analysis centres on English primary care and in particular on the issue of how healthcare professions are affected by, and in turn affect, the interpretation and adoption of new services. We use the case of the implementation of evidence-based approaches for managing patients with osteoarthritis. This musculoskeletal problem occurs in a high proportion of GP consultations, and is projected to increase due to a rapidly ageing population in the western world’ [29].
	**Adaptive execution** [10]	**Description:** How do contexts affect the ways in which users can find and enact workarounds that make an intervention and its components a workable proposition in practice? [11]. **Example:** ‘Huge effort was expended and continues to be required to implement and keep this technology in use. This innovation must be understood both as a computer technology and as a set of practices related to that technology, kept in place by a network of actors in particular contexts. While technologies can be ‘made to work’ in different settings, successful implementation has been achieved, and will only be maintained, through the efforts of those involved in the specific settings and if the wider context continues to support the coherence, cognitive participation, and reflective monitoring processes that surround this collective action. Implementation is more than simply putting technologies in place – it requires new resources and considerable effort, perhaps on an on-going basis’ [30].
	**Negotiating capacity** [10]	**Description:** How do contexts affect the extent that an intervention and its components can fit, or be integrated, into existing ways of working by their users? [11]. **Example:** ‘Aligning IPC guidelines with local clinical context is an essential means to reduce the sense of dissonance and represents a critical step forward towards successful implementation. Some strategies described in the literature to promote alignment include: integration of IPC recommendations within other established programmes; and education and audit interventions acknowledging the positive and negative beliefs of staff on IPC practices [31].
	**Reframing organisational logics** [10]	**Description:** How do existing social structural and social cognitive resources shape the implementation environment? [11]. **Example:** ‘The external and internal partnership building were key and also strategic, so as not to impose ERAS but to co-create it from the ground up. This relational work, as framed in the NPT, is deceptively complex as it involves convincing others that this is a legitimate improvement programme worth participating in without devaluing their current practice and beliefs. The interprofessional and interdepartmental relationships the champion teams established appeared to lay an important foundation for accepting changes and the data reports as meaningful and embedding ERAS into everyday practice’ [32].
Implementation mechanisms: Mechanisms are revealed through purposive social action—**collaborative work**—that involves the investment of personal and group resources to achieve goals	**Coherence building** [7]	**Description:** How do people work together in everyday settings to understand and plan the activities that need to be accomplished to put an intervention and its components into practice? [11]. **Example:** ‘Coherence was achieved around the CDSS despite local context variation. Across all three sites there was agreement that the CDSS was suitable for the (varied) tasks and that appropriate resources were in place to enable effective implementation, although these varied between settings. There were differences between settings where the CDSS replaced an established system with existing staff and where the service and/or the staff were new and the work of establishing coherence had to be altered to reflect this. It was clear that knowledge, experience and work identities built through doing call-handling work influenced the coherence of the CDSS for staff in the different settings. What is especially interesting in the wider policy context – where this same CDSS is now being used to support a national ‘111’ urgent care service (...) is that coherence was not just a local ‘problem’, it was necessarily underpinned by wider understandings and discourses for example about the necessity of rationing and the need to modify caller/patient behaviour and beyond that the very legitimacy of evidence based medicine and the kinds of expert knowledge which underpinned the CDSS’ [30].
	**Cognitive participation** [7]	**Description:** How do people work together to create networks of participation and communities of practice around interventions and their components? [11] **Example:** ‘Cognitive participation relates to the work that participants undertake to build up and sustain a community of practice around an intervention. In terms of CST, participants identified training as an important factor in generating their own and their colleagues’ interest in CST and thus ensuring all stakeholders were involved. Staff were further motivated to continue running the groups within their service through observing the direct beneficial effects of CST on clients’ [33].
	**Collective action** [7]	**Description:** How do people work together to enact interventions and their components? [11]. **Example:** ‘The daily tasks involved in carrying out Point of Care (POC) testing were deciding which tests (if any) to take for each patient when they arrived; communicating this to others; taking the blood; running the tests; examining the results; communicating the results to others; and deciding what action to take accordingly. This work was allocated to different staff according to their skills and availability. Close teamwork appeared key to ensuring that each task was performed by an appropriate person at the necessary time’ [34].
	**Reflexive monitoring** [7]	**Description:** How do people work together to appraise interventions and their components? [11]. **Example:** ‘Data provision by the laboratories proved to be difficult despite the standardized format. The database manager at the central level reported he had to put much effort in getting the data from the system administrator from the laboratories because they did not prioritize data delivery. It was reported by them that saving the data extraction queries, as the research group suggested, for use in the next time period was increasingly helpful in the course of the implementation period. By fine-tuning these queries after each extraction, the quality of the delivered data improved’ [35].
Implementation outcomes: The practical effects of implementation mechanisms at work	**Intervention performance** [6]	**Description:** What practices have changed as the result of interventions and their components being operationalized, enacted, reproduced, over time and across settings? [11] **Example:** ‘The bed-monitoring technologies were felt to be useful in helping staff identify patterns in resident behaviour and explore reasons for these behaviours. The bed sensors at Sycamore Lane were capable of recording clinical data such as heart rate, but the manager reported that “it’s not something that we use readily”, and this functionality was never observed in use during the present study. The location-based system at Conifer Gardens was similarly able to record data, including information about resident mobility activity. This functionality had initially been anticipated as potentially useful for enhancing clinical understanding, however, the Occupational Therapist reflected that the time needed to analyze and interpret these data had been “a job in itself” and thus has been difficult to integrate into daily practice. There were questions about the clinical utility of some of the data, which appeared to become more pronounced when considering the financial expense of the technology’ [36].
	**Relational restructuring** [10]	**Description:** How have working with interventions and their components changed the ways people are organized and relate to each other? [11]. **Example:** ‘The CMs became “everyday representatives” for the secondary sector and were responsible for acting as bridge- builders between hospital psychiatry and general practice. Previous research on Nurse Practitioners/ Advanced Nurse Practitioners in general practice (...) has shown that if the clinics are not involved at an early stage and prepared thoroughly for the Nurse Practitioner's arrival, their integration in general practice is hampered. Preparation involves practical issues, a clearly defined role for the nurse practitioner, and organizational leadership, meaning that the managers of the responsible organization must be involved in the process of defining and supporting the role (...) The challenges also pointed towards a lack of managerial co-ordination of, and responsibility for, the practical issues associated with the CM's role in general practice. (...) This meant that on many occasions, the CMs had to take on the role of implementation ambassadors assuming responsibility for maintenance of the collaborative care model’ [37].
	**Normative restructuring** [10]	**Description:** How have working with interventions and their components changed the norms, rules and resources that govern action? [11]. **Example:** ‘The first theme, trusting and embedding new relationships, is a reminder that while locally-led innovation is designed to address local problems, convincing others of its value is core work. This is particularly so when the innovation challenges professional norms and involves changes to traditional delivery models and renegotiation of professional roles (...). In this case, the findings are consistent with previous research which has indicated that the success of such innovations is dependent on the trust of all involved and the credibility of clinicians (...)’ [38].
	**Sustainment (normalisation)** [6]	**Description:** How have interventions and their components become incorporated in practice? [11]. **Example**: ‘At the end of the project period, the pathway was integrated in daily practice in two of the six municipalities. In these municipalities the care pathway was found to have the potential of structuring the provision of home care services and collaboration with the GPs, and serving as a management tool to effect change and improve knowledge and skills. (...) The generic care pathway for elderly patients has a potential of improving follow-up in primary care by meeting professional and managerial needs for improved quality of care, as well as more efficient organization of home care services. However, implementation of this complex intervention in full-time running organizations was demanding and required’ [39].

**Table 2:** NPT coding manual part B: secondary constructs—mechanisms

NPT construct	Sub-construct	Description and example
**Coherence:** How do people work together to understand and plan the activities that need to be accomplished to put an intervention and its components into practice? [11].	**Differentiation** [7]	**Description:** How do people distinguish interventions and their components from their current ways of working? [40]. **Example:** ‘In order to invest in ERAS individuals needed to be able to differentiate its practices favourably with those enacted pre-implementation. This required *coherence* work in understanding the potential patient benefits allied to its introduction. Participants provided divergent accounts when they compared ERAS to previous practice. A number of participants asserted that the introduction of ERAS had brought about considerable changes to their day-to-day practice. These changes included positive adjustments in the management of patients and required patients to play a more active role in their own recovery’ [32].
	**Communal specification** [7]	**Description:** How do people collectively agree about the purpose of interventions and their components? [40]. **Example:** ‘Another barrier to coherence was lack of communal specification, since not everyone considered they had been informed about the study or understood its aims and processes. This caused implementation problems for the homes and the research team. For the homes, the researchers’ reasons for examining potential benefits from the intervention to have a positive impact on the culture of care had not been strongly reflected’ [41].
	**Individual specification** [7]	**Description:** How do people individually understand what interventions and their components require of them? [40]. **Example**: ‘One respondent felt discussing the new way to view the patients with the staff was a delicate issue. In the old care model, patients were usually only informed about the treatment whilst now, in the care model, patients were to be seen as partners. This was regarded as a shift in power and, at least for some physicians, it would be difficult to get used to’ [42].
	**Internalisation** [7]	**Description:** How do people construct potential value of interventions and their components for their work? [40]. **Example**: ‘At this stage (initial introductory meetings), the value of the intervention was purely based on individuals’ interpretation of the information given by the research team and the “fit” with their own interests. The GPs in General Practice 8 provided their views at the end of the introductory meeting, saying that they liked the structure and more systematic approach to caring for people with OA and concluded that “it is nice to be able to try something that may make a difference”’ [43].
**Cognitive participation:** How do people work together to create networks of participation and communities of practice around interventions and their components? [11]	**Initiation** [7]	**Description:** How do key individuals drive interventions and their components forward? [40] **Example**: ‘Participants described the new SDM work as requiring leaders to define the work, and then enrolling others to contribute collectively to the process. Identifying leadership support for SDM was challenging: clinical teams are not simple hierarchical units, and substantial autonomy exists, especially for experienced clinicians’ [44].
	**Enrolment** [7]	**Description:** How do people join in with interventions and their components? [40]. **Example**: ‘Clinic participants also re-ported that the intervention provided a model for improved interprofessional team collaboration, resulting in a greater understanding of clinicians’ roles and skill sets. Huddles were viewed as worth creating and maintaining, both for interprofessional team and patient benefits. Participants identified that the majority of patients were satisfied with the interprofessional approach to primary care’ [45].
	**Legitimation** [7]	**Description:** How do people agree that interventions and their components are the right thing to do and should be part of their work? [40]. **Example**: ‘The respondents offered several explanations for resistance or lack of engagement: some staff felt that health promotion activities overstretched users’ resources and thus had a negative impact on their quality of life; others argued that health promotion activities did not respect personal preferences of users and staff (…) One of the important implementation ideas (…) was the concept of staff being role models for health promotion. As role models staff were[ expected to participate in different health promotion activities (like joining users for walks and meals) and to display a healthy lifestyle at work. In the four providers, such expectations were formulated and formalised by management or by key implementation staff to different extents. However, in all cases some staff did not buy into this idea; they felt that the elements of smoking cessation and healthier meals interfered with their usual lifestyle and personal preferences’ [46].
	**Activation** [7]	**Description:** How do people continue to support interventions and their components? [40]. **Example**: ‘While, overall, this system has worked well, many participants referenced instances of long wait times and rerouting of calls to reach the neonatologist. Based on the care teams' appraisal and experience with this process, they suggested modeling the teleneonatology service activation after the emergency department's response system, for immediate and direct connection. Other suggestions include making the technology simple enough for ease of use, and to mount a camera (which can be controlled by the remote neonatologist) to the baby warmer’ [47].
**Collective action:** How do people work together to enact interventions and their components? [11].	**Interactional workability** [7]	**Description:** How do people do the work required by interventions and their components? [40]. **Example**: ‘The rural allied health team indicated that telehealth technology provided ‘a whole range of other capabilities’, and considered it ‘safe and it’s appropriate and it’s an equivalent, if not better, sort of service that you can provide’. They were committed to the notion that telehealth could balance the unequal access to services across geographical locations, and were keen to pursue innovative ways of using telehealth technologies to allow them to provide complex distant therapy. In contrast to rural and experienced telehealth clinicians who were keen to utilise technology as part of their role and to deal with distance and isolation, urban clinicians with no exposure to telehealth reported more reservations about the safety and suitability of providing rehabilitation through telehealth. They generally felt that telehealth should be reserved for ‘people who are more autonomous and more capable and … straightforward’, rather than ‘real’ rehabilitation patients with complex issues. They felt that people who required rehabilitation often require a ‘hands on’ approach’ [48].
	**Relational integration** [7]	**Description:** How does using interventions and their components affect the confidence that people have in each other? [40]. **Example**: ‘Enhanced collegial discussion about FV and adherence to the safety measures, such as the home visiting policy and procedures introduced in (…) model, were important for nurses to feel safe and undertake the FV work. As implementation progressed, intervention nurses felt safer than comparison nurses when attending home visits (…). Relationships within teams and with FV services varied across the MCH intervention teams. High workloads, time constraints and a lack of nursing staff or relievers in some centres impacted on the organisation of the FV work at times. The nurse mentor role to provide secondary consultation, linkage to FV services and support for other MCH nurses had varied success. Due to time constraints and the often solo nature of MCH practice, most nurses preferred to discuss clinical issues with a nurse friend or co-worker at the time rather than try to contact the designated MOVE nurse mentor, with only 38% of nurses using the nurse mentor role early in the trial. This increased to 52% as time went on. If the nurse was not comfortable speaking and had insufficient time or access to the nurse mentor, then this aspect of the model was lost’ [49].
	**Skill-set workability** [7]	**Description:** How is the work of interventions and their components appropriately allocated to people? [40]. **Example**: ‘A key theme identified in the literature and through this study is the need for more training for practitioners. This includes training both in professional education and continuing educational opportunities for all practitioners. Medical, nursing and allied health education programs need to improve LGBT curriculum content (…). Providing education on general terminology, healthcare needs specific to the transgender population, and practitioners’ role in providing healthcare for this population will better prepare new practitioners for serving this community. Increased access to continuing education with LGBT content will help to increase the knowledge and skill of current practitioners. Embedding LGBT content within current programs of continuing education may increase awareness more than having specific LGBT courses (…). Embedding it in current programs may bring awareness to the concepts and highlight the need for practitioners to seek out more specific training to address their learning gaps’ [50].
	**Contextual integration** [7]	**Description:** How is the work of interventions and their components supported by host organizations? [40]. **Example**: ‘Since POs were able to self-select into the pilot, the alignment of PO priorities with participation in a pilot on care management was a good fit. The leadership in all POs voiced interest in providing care management to patients within their PO as a means of improving patient outcomes, easing burden on providers of handling complex patients, and to meet health care standards and reimbursement policies such as patient-centred medical home recognition, accountable care, and meaningful use. Therefore, in this study overall organizational support was not found to be variant. Where organizational support emerged as an issue related more to resources and support for the care management program relative to the needs and goals of the program. The most common issue here was not having either enough care managers or enough care manager protected time to do care management for the number of patients needing it. So in well-normalized programs, there was a sense of “rationing” of the care manager. Because the program was being used so much more and there was a capacity constraint at the practice level with the practice-based care manager structure, the practices in these POs voiced more concern about lack of care manager capacity. Lack of resources was evident in other ways such as lack of space for patient visits or access to phone lines to make longer calls’ [51].
**Reflexive monitoring** **Description:** How do people work together to appraise interventions and their components? [11].	**Systematisation** [7]	**Description:** How do people access information about the effects of interventions and their components? [40]. **Example**: Feedback was never provided to staff on the effect of the AKI e-alert “I haven’t had any feedback since the new version (of the AKI e-alert) went in actually(...) I don’t know whether there is a formal mechanism for that getting to anyone”’ [52].
	**Communal appraisal** [7]	**Description:** How do people collectively assess interventions and their components as worthwhile? [40]. **Example**: ‘The e-alert was rarely (if ever) discussed among clinicians, but participants often stated they felt that others would find it worthwhile. “The e-alert was rarely (if ever) discussed among clinicians, but participants often stated they felt that others would find it worthwhile. “Most people I'm sure would know it's a good idea having them. That's what I'd say to someone about these alerts”’ [52].
	**Individual appraisal** [7]	**Description:** How do people individually assess interventions and their components as worthwhile? [40]. **Example**: ‘A key barrier which has not previously been identified concerned the ability of case managers to identify, and act on, emerging patient and carer needs; we identified examples of missed and unmet needs for all three case managers. One case manager explicitly attributed this to the timing of the intervention; a study of case management for people with early symptoms of dementia and their carers similarly found that case managers did not feel the intervention was needed at this point’ [53].
	**Reconfiguration** [7]	**Description:** How do people modify their work in response to their appraisal of interventions and their components? [40]. **Example**: ‘Aligning IPC guidelines with local clinical context is an essential means to reduce the sense of *dissonance* and represents a critical step forward towards successful implementation. Some strategies described in the literature to promote alignment include: integration of IPC recommendations within other established programmes; and education and audit interventions acknowledging the positive and negative beliefs of staff on IPC practices’ [31].

## Results: a coding manual for normalisation process theory

**Fig. 1:**
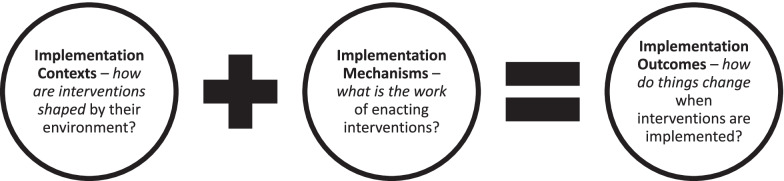
Linking NPT to realist evaluation: implementation contexts, mechanisms, and outcomes

Working through the procedures described above led to part A of the coding manual for NPT. This is presented in Table 1 and consists of 12 primary NPT constructs. Although it was not originally intended to do so, we found that the final structure of the coding manual sits easily alongside the Context-Mechanism-Outcome configuration of realist evaluation studies [54]. We describe this in Fig. 1. The array of primary NPT constructs took the following form.

1. *Contexts* are events in systems unfolding over time within and between settings in which implementation work is done (primary NPT constructs: strategic intentions, adaptive execution, negotiating capability, reframing organisational logic).
2. *Mechanisms* motivate and shape the work that people do when they participate in implementation processes (primary NPT constructs: coherence-building, cognitive participation, collective action, reflexive monitoring).
3. *Outcomes* are the effects of implementation work in context—that make visible how things change as implementation processes proceed (primary NPT constructs: intervention performance, normative restructuring, relational restructuring, sustainment).

These 12 constructs form a general set of codes that can be applied to almost any textual data whether these are fieldnotes, interview transcripts, or published texts. They are all grounded in empirical research. Part A (Table 1) of the coding manual thus provides a general set of codes or categories that guide analytic work. Each construct is named and briefly described. Additionally, each descriptor is accompanied by an example of the empirical application of the construct in already published work. The aim of a coding manual is to provide guidance about how to interpret data that is often highly nuanced and represents complex and sometimes very dynamic processes at work. However, no theory, framework, or model can generate a set of codes that will infallibly cover all possible features of data. In this case, guidance about interpretation, rather than scriptural authority, is the primary intention of our coding manual. Detailed guidance on the process of coding can be found in work by Strauss [1] and Schreier [2].

More granular possibilities are presented in part B (Table 2) of the coding manual. Here, the four NPT primary constructs related to *mechanisms* of purposive social action (coherence-building, cognitive participation, collective action, reflexive monitoring), each possess four associated secondary constructs. These secondary constructs provide further and equally empirically grounded codes where the available qualitative data support interpretation at that level of detail. Once again, each construct is named and briefly described, and each descriptor is accompanied by an example of the empirical application of the construct in already published work. However, the use of these 16 secondary constructs in coding is not mandatory, and many papers included in systematic reviews [12–14] of NPT studies seem either to have treated them as discretionary or not referred to them at all. They are however valuable and important, and thus have explanatory value, because the mechanisms that motivate and shape implementation processes are often those that are mobilised to *overcome* perceived problems of context. In NPT, analysis always focuses on purposive social action—the *work* that people do to enact evidence or innovation in practice—and for this reason, focusing attention on the constructs that characterise action is central to the interpretive task.

## Discussion

The purpose of developing this coding manual was to clarify and simplify NPT for the user and to make it more easily integrated and workable in research on the adoption, implementation, and use of sociotechnical and organisational innovations. In qualitative content analysis—as in other forms of qualitative analysis—proliferating constructs can easily make the business of coding ever more microscopic and can mean that it becomes less analytically rewarding. Indeed, the more parsimonious a prescheduled theoretical structure is, the more space it provides for nuanced interpretation and the development of novel categories of data and the analytic constructs that can be derived from them.

In the development of the NPT coding manual described here, we sought to eliminate ambiguity and add workability from the outset. The process of selection and structuring we describe yielded a set of 12 primary NPT constructs (Table 1: coding manual part A) and 16 sub-constructs (Table 2: coding manual part B). As Fig. 1 shows, these identify, characterise, and explain the course of implementation processes through which strategic intentions are translated into practices and enable understanding of how enacting those practices can lead to different outcomes, and to varying degrees of sustainment.

Coding is a centrally important procedure in qualitative analysis [1], but it must be emphasised that it is only one part of a whole bundle of cognitive processes through which researchers make and organise meanings in the data. Here, a coding manual cannot cover all analytic possibilities presented by a qualitative data set. Reflexive procedures for identifying phenomena outside the scope of a theory, developing new codes, and linking them to other explanatory models are always important in theory-informed qualitative work. The act of coding involves descriptive work that is a foundation for the interpretation of data, but it is not a proxy for it nor is the purpose of a coding manual to verify the underpinning theory. The whole purpose of coding, and of linking coding to theory, is to build and inform interpretation and understanding. This is not a discrete stage in data analysis but is continuous throughout [1].

Linking NPT to the CMO model of realist evaluation did not happen by accident. NPT was developed through a series of iterations that were already heading in this direction. This began with empirical studies that led to rigorous analysis of the *mechanisms* that motivate and shape implementation processes [7, 8]. As the theory was developed and applied, further consideration was given to the problem of *contexts* [9, 13, 16] and to the question of how mechanisms interact with contexts to produce specific *outcomes* [11, 24, 25, 55]. At the same time, systematic reviews [12–14] revealed that the use of NPT was impeded because researchers without a strong theoretical background in the social sciences needed both clearer definitions of constructs and a conceptual toolkit that linked these together in a way that enabled them to see how implementation mechanisms and contexts interact with each other to shape different kinds of outcomes. Drawing these together in a single-coding manual would assist in solving these problems.

### Strengths and limitations

We describe a set of methods likely to be useful be useful to qualitative researchers in other areas of research who wish to consider developing such manuals for other theories (for example, relational inequalities theory [56] or event system theory [57]). A strength of the work was that developing the coding manual was undertaken by an international multidisciplinary team working with personal experience of developing and working with NPT and with other implementation frameworks, models, and theories. This ensured that from the outset the development of the coding manual was closely linked to knowledge about the ways that NPT can be used. An unanticipated consequence of the coding manual being published on preprint servers (ResearchSquare.Com and ResearchGate.Com) was that other researchers started to use it almost immediately and quickly fed back criticism or encouragement. This added value to both the development process and the final product.

This work was undertaken opportunistically and grew organically. The manual thus developed cumulatively and in an ad hoc way. Working from a structured protocol would have added greater methodological transparency and perhaps also potential for replication of the development process. Finally, researchers working from different perspectives, with different experiences of NPT, using primary empirical studies rather than theory papers, or working from a prescheduled protocol, might have produced a different coding manual.

## Conclusion

This paper describes the procedures by which the NPT coding manual for qualitative research was produced. It also presents the manual ready for use. But more than this, the process of producing the coding manual has also led to the simplification and consolidation of the theory by bringing together empirically grounded constructs derived from multiple iterations of theoretical development over two decades.

Coding manuals are useful tools to support analysis in qualitative research. They reduce cognitive load and at the same time render the assumptions underpinning qualitative analysis transparent and easily shared amongst teams of researchers. The coding manual makes the application of NPT simpler for the user. This adds value to qualitative research on the adoption, implementation, and sustainment of innovations by providing a stable, workable, set of constructs that sit comfortably alongside the well-established model of realist evaluation [54]. It also forms a translational framework for researching and evaluating implementation processes and thus complements other resources for NPT researchers such as the NPT Toolkit and the NOMAD survey instrument [17, 19, 21, 22].

## Supplementary Information

**Additional file 1.**

## Data Availability

All materials used in this research are included in this paper as tables or are appended as online supplementary materials.

## Abbreviations

AKI: Acute kidney injury
CDSS: Computer decision support system
CM: Care manager
CST: Cognitive stimulation therapy
ERAS: Enhanced recovery after surgery
FV: Family violence
GP: General practice
IPC: Infection prevention and control
LGBT: Lesbian, gay, bisexual and transsexual
MCH: Mother and child health nurse
MOVE: Improving Maternal and Child Health Care for Vulnerable Mothers (Trial Acronym)
NPT: Normalisation process theory
OA: Osteo-arthritis
PO: Physician organisations
SDM: Shared decision-making
