# Supplementary material for: Translational framework for implementation evaluation and research: a normalisation process theory coding manual for qualitative research and instrument development
Source: Implement Sci. 2022 Feb 22;17:19. doi: 10.1186/s13012-022-01191-x (PMC8861599; doi:10.1186/s13012-022-01191-x)
Supplement: Supplementary file 1 — Additional file 1. [file 13012_2022_1191_MOESM1_ESM.docx]

**Evaluating Implementation Contexts, Mechanisms, and Outcomes:**

**a Normalization Process Theory coding manual for qualitative research and instrument development**

**ONLINE SUPPLEMENTARY MATERIAL**

**Carl R May** [Carl.May@lshtm.ac.uk](mailto:Carl.May@lshtm.ac.uk) Department of Health Services Research and Policy, London School of Hygiene and Tropical Medicine & NIHR North Thames ARC

**Bianca Albers** [bianca.albers@uzh.ch](mailto:bianca.albers@uzh.ch) Institute for Implementation Science in Healthcare, Zurich, Switzerland

**Mike Bracher** [M.J.Bracher@soton.ac.uk](mailto:M.J.Bracher@soton.ac.uk) School of Health Sciences, University of Southampton

**Tracy L Finch** [tracy.finch@northumbria.ac.uk](mailto:tracy.finch@northumbria.ac.uk) Department of Nursing, Midwifery & Health, Northumbria University & NIHR ARC North East-North Cumbria, Newcastle, UK

**Anthony Gilbert** [anthony.gilbert@nhs.net](mailto:anthony.gilbert@nhs.net) Royal National Orthopaedic Hospital, London & NIHR North Thames ARC

**Melissa Girling** [melissa.girling@northumbria.ac.uk](mailto:melissa.girling@northumbria.ac.uk) Department of Nursing, Midwifery & Health, Northumbria University & NIHR ARC North East-North Cumbria

**Kathryn Greenwood** [k.e.greenwood@sussex.ac.uk](mailto:k.e.greenwood@sussex.ac.uk) School of Psychology, University of Sussex

**Anne MacFarlane** [Anne.MacFarlane@ul.ie](mailto:Anne.MacFarlane@ul.ie) School of Medicine and Health Research Institute, University of Limerick, Ireland

**Frances S Mair** [Frances.Mair@glasgow.ac.uk](mailto:Frances.Mair@glasgow.ac.uk) Institute of Health and Wellbeing, Glasgow University

**Christine M May** [chrismay918@gmail.com](mailto:chrismay918@gmail.com) Independent Researcher, Southampton, UK

**Elizabeth Mur**ray [elizabeth.murray@ucl.ac.uk](mailto:elizabeth.murray@ucl.ac.uk) Research Department of Primary Care and Population Health, University College London & NIHR North Thames ARC

**Sebastian Potthoff**. [sebastian.potthoff@northumbria.ac.uk](mailto:sebastian.potthoff@northumbria.ac.uk) Department of Social Work, Education and Community Wellbeing, Northumbria University & NIHR ARC North East- North Cumbria

**Tim Rapley** [tim.rapley@northumbria.ac.uk](mailto:tim.rapley@northumbria.ac.uk) Department of Social Work, Education and Community Wellbeing, Northumbria University & NIHR ARC North East-North Cumbria

**EXPLANATORY NOTE**

To develop the coding frame, all identifiable theoretical constructs (n=149) embedded in papers and chapters that developed NPT between 2006 and 2020 were identified and extracted from their texts. These are presented in Table 1, below. Then, overlapping, ambiguous, and duplicate versions of constructs were eliminated, as were constructs derived from other theories. This left 38 core concept definitions. These are presented in Table 2, below.

**Table 1. Theoretical constructs extracted from NPT papers published between 2006-2020**

|  | *Actors* are the individuals and groups that encounter each other in health care settings. [1, 2] |
| --- | --- |
|  | *Allocation* is concerned with which tasks are performed by whom and how these decisions are made, the distribution of resources and rewards linked to status and authority, formal or informal agreements about the identification and appraisal of the necessary skills, and the definition and ownership of these skill-sets. [1, 2] |
|  | *Co-operative* attributes that are oriented towards enacting the intervention through negotiations and agreements between people and the organizations and policymakers providing the context within which they work[1, 2] |
|  | *Complex intervention* is defined as a deliberately initiated attempt to introduce new, or modify existing, patterns of collective action in health care. [1, 2] |
|  | Complexity: the situations in which complex interventions are initiated are, of course, contingent, variable, dynamic and unevenly distributed. [1, 2] |
|  | *Confidence* refers to agreement about the sources of authoritative knowledge and practice, the criteria by which their credibility can be assessed, and beliefs about the practical utility and reliability of the knowledge and practice mediated by the various networks in the health system. [1, 2] |
|  | Confounders that include complexity and emergence that lead to local variations in implementation processes. These confounders may include events or processes far beyond the purview of participants in the implementation of a complex intervention. Importantly, they include many external factors that are not amenable to control or modification. [1, 2] |
|  | *Contexts* are the physical, organisational, institutional, and legislative structures that enable and constrain, and resource and realize, people and procedures. [1, 2] |
|  | *Contextual Integration* – how does a complex intervention relate to the organisation in which it is set? [1, 2] |
|  | De-normalization may also occur during the lifetime of a complex intervention when a previously normalized intervention is superseded, disturbed, disrupted, or atrophied. Thus, normalization is neither an automatic outcome nor a permanent state. [1, 2] |
|  | *Deliberate initiation* means that an intervention is: institutionally sanctioned; formally or informally defined; consciously planned; and intended to lead to a changed outcome. [1, 2] |
|  | Embedding by reference to social processes. These processes are located in specific contexts and take place over time (that is, those who initiate them have an end in sight). [1, 2] |
|  | Emergent qualities of implementation processes mean that empirical rather than theoretical investigation is a vital part of the development of the model. Coherence, consistency and explanatory power therefore need to be maintained by establishing the proper range and scope of the theory and staying within it. [1, 2] |
|  | *Execution* is concerned with the practicalities of integration (e.g. does the intervention require new money, a local or national policy sponsor), decisions about the distribution of resources, costs and risks within the organisation, managerial decision-making regarding the adoption of the intervention, and formal and informal mechanisms for its evaluation. [1, 2] |
|  | *Executive* attributes that are oriented to attempts to project enacting the intervention outwards in time and space. [1, 2] |
|  | Implementation. This is defined as a pattern of organized, dynamic, and contingent interactions in which individuals and groups work with a complex intervention, within a specific context or health system, over time. [1, 2] |
|  | *Initiators* of a complex intervention may seek to change the ways that people think, act and organize themselves in health care, or they may seek to initiate a process with the intention of creating a new outcome. (i) [1, 2] |
|  | *Interactional Workability* – how does a complex intervention affect interactions between people and practices? [1, 2] |
|  | Normalization as an *accomplishment*, and normalization as a possible *outcome* of that accomplishment. [1, 2] |
|  | Normalization process consists of the collective action – the *work* – involved in enacting a complex intervention. [1, 2] |
|  | Normalized: When that work leads to the routine embedding of an intervention in everyday practice, it may be said to have become normalized. Normalization does not, however, imply an evaluation of effectiveness or quality. [1, 2] |
|  | *Objects* are the institutionally sanctioned means by which knowledge and practice are enacted. [1, 2] |
|  | O*rganizational* processes characterized by complexity and emergence, where multiple confounders act upon behaviour, experienced ingroup processes in structured organizational contexts. [1, 2] |
|  | *Other theories: Diffusion and adoption:* The diffusion of innovations across networks of organizations or organizational units, and their adoption by individual or collective 'champions' is the proper domain of diffusion of innovations theory. [1, 2] |
|  | *Other theories: Intention and volition:* The mental components of individual behaviour, especially the cognitions and intentions that might dispose individuals to adopt a complex intervention, are the proper domain of psychological theory. [1, 2] |
|  | *Performance* considers the ability of an organisation and the people within it to effectively organise and deploy a complex intervention as part of their activities. It covers staff training needs, formal and informal policies that define the boundaries of competence of particular workers, the degree of autonomy these assigned to them, and how they deliver services. [1, 2] |
|  | *Phenomena* that are the products of co-operative and collective activities, [1, 2] |
|  | *Processes* by which complex interventions are made *workable* and *integrated* in everyday practice. [1, 2] |
|  | *Realisation* considers the allocation and ownership of responsibility for the implementation of a complex intervention (e.g. does the complex intervention require responsibility for a procedure to move from one professional group to another?), the negotiations necessary to modify existing systems and practices to make new ones possible, minimising the disruption and risk associated with change, and how new resources are obtained and used in practice. [1, 2] |
|  | *Relational Integration* – how does a complex intervention relate to existing knowledge and relationships? [1, 2] |
|  | *Skill-set Workability* – how is the current division of labour affected by a complex intervention? [1, 2] |
|  | *Work* as collective action, over time. [1, 2] |
|  | Implementation, by which we mean the social organization of bringing a practice or practices into action.[3, 4] |
|  | Embedding, by which we mean the processes through which a practice or practices become, (or do not become), routinely incorporated in everyday work of individuals and groups. [3, 4] |
|  | Integration, by which we mean the processes by which a practice or practices are reproduced and sustained among the social matrices of an organization or institution. [3, 4] |
|  | Practices become routinely embedded – or normalized – in social contexts as the result of people working, individually and collectively, to enact them. [3, 4] |
|  | Work of enacting a practice is promoted or inhibited through the operation of generative mechanisms (coherence, cognitive participation, collective action, reflexive monitoring) through which human agency is expressed. [3, 4] |
|  | Production and reproduction of a practice requires continuous investment by agents [3, 4] |
|  | Empirical generalizations treated 'normalization' as the endpoint of an implementation process in which some new technology came to be routinely employed in service. [3, 4] |
|  | Coherence: Work that defines and organizes the objects of a practice. [3, 4] |
|  | Cognitive participation: Work that defines and organizes the enrolment of participants in a practice. |
|  | Collective action: Work that defines and organizes the enacting of a practice. [3, 4] |
|  | Reflexive monitoring: Work that defines and organizes the knowledge upon which appraisal of a practice is founded. [3, 4] |
|  | Material practices become routinely embedded in social contexts as the result of people working, individually and collectively, to implement them. From this follows specific propositions that assert that define a mechanism (*i.e.*, embedding is dependent on socially patterned implementation work). [3, 4] |
|  | Implementation as a social process of collective action [5] |
|  | Implementation processes as interactions between ‘emergent expressions of agency’ *(i.e*., the things that people do to make something happen, and the ways that they work with different components of a complex intervention to do so); [5] |
|  | Implementation as ‘dynamic elements of context’ (the social-structural and social-cognitive resources that people draw on to realize that agency). [5] |
|  | Agentic contributions and capability, and the potential and capacity for resource mobilization. [5] |
|  | Agency *(i.e*., their ability to make things happen through their own actions). [5] |
|  | Implementation as a process: Agents seek to make these processes and contexts plastic: for to do one thing may involve changing many others. Implementation therefore needs to be understood from the outset as a process – that is, as a continuous and interactive accomplishment – rather than as a final outcome. [5] |
|  | Implementation’ never refers to a single ‘thing’ that is to be implemented. Whenever some new way of thinking, acting, or organizing is introduced into a social system of any kind, it is formed as a complex bundle – or better, an ‘ensemble’ – of material and cognitive practices. [5] |
|  | Social system is defined as a set of socially organized, dynamic and contingent relations. These relations form a structure that is populated by agents (who may be individuals or groups) that interact with each other. Information and other resources flow through these interactions between agents. [5] |
|  | System therefore forms structural conditions for the expression of agency. [5] |
|  | Social systems are emergent, which means that they are shaped, over time and across space, by both endogenous and exogenous factors. This means that their future is relatively unpredictable. [5] |
|  | Mechanisms operate. In this paper, a mechanism is defined as a ‘process that brings about or prevents some change in a concrete system’, that ‘unfold[s] over time’, and expresses contributions of human agency. [5] |
|  | Agents jointly construct their own actions as pragmatic, strategic responses to their circumstances and as expressions of commitment to their values’. In this context, a mechanism-based approach focuses on the things that agents do to make their affairs plastic or malleable. [5] |
|  | Emergence in social systems and plasticity in social mechanisms mean that the future shape and form of any social process is uncertain. of science and technology. [5] |
|  | Implementation can be characterized as a deliberately initiated process, in which agents intend to bring into operation new or modified practices that are institutionally sanctioned, and are performed by themselves and other agents [5] |
|  | Agents – who are the individuals and groups that encounter each other in healthcare settings – engage in the realization and mobilization of material and cultural resources, and secure the consent, cooperation and expertise of those other agents who inhabit the particular field or domain of action in which the process of implementation takes place [5] |
|  | Implementation subsumes all related activities from initiation to incorporation [[37](https://link.springer.com/article/10.1186/1748-5908-8-18#ref-CR37)], and it may lead to the routine incorporation of ensembles of practice in everyday work. [5] |
|  | Object of an implementation process is subsumed under the ambit of a ‘complex intervention’ – a cognitive and behavioral ensemble that involves different material and cognitive practices, relations and interactions. [5] |
|  | Agents engage with complex interventions, they engage with multiple objects of practice. These may include classifications, real or virtual artifacts and techniques, technologies or organizational systems. [5] |
|  | Attributes of the components of complex interventions themselves affect their use. Such attributes include their virtual or physical character, the assumptions about use and users that are embedded within them, their complexities in practice and in the social relations that they engender, and their expected value. [5] |
|  | Components All of these elements combine to make them much more than the sum of their parts and to shape the relations between agents and the different components of a complex intervention through processes of mutual co-constitution. [5] |
|  | The qualities of complex interventions – whether they are workable in, and can be integrated into, practice – are therefore important elements of implementation processes. [5] |
|  | Material practices that agents perform when they operationalize a complex intervention (its interactional workability), and the ways in which these practices were linked to, and distributed through, a division of labor (its skill set workability). [5] |
|  | Integration can be divided into contextual integration, in which the performance of a practice is linked to the means by which it is realized and to the resources transmitted to it, and relational integration, in which the performance of a practice is linked to the means by which users make themselves and others accountable for its performance. [5] |
|  | Users make objects workable through use, an [5]d they work to integrate them in their social contexts. [5] |
|  | Workability: the social practices that agents perform when they operationalize a complex intervention within a social system, and characterizes interactions between users and components of a complex intervention; [5] |
|  | Integration: the linkages that agents make between the social practices of a complex intervention and elements of the social system in which it is located, and characterizes interactions between the context of use and components of a complex intervention. [5] |
|  | Object of an implementation process is some new or modified way of thinking, enacting or organizing action. [5] |
|  | Object may be virtual or concrete, or both, and it is always associated with an ensemble of cognitive and behavioral practices. It can thus be characterized as a complex intervention, and the possibilities it presents to agents [5] |
|  | Normalization complex intervention is disposed to normalization into practice if its elements, and their associated cognitive and behavioral ensembles can be made workable and integrated in everyday practice by agents. If workability and integration cannot be sustained, then the embeddedness of the complex intervention will be threatened as the capacity of agents to employ it is confounded. [5] |
|  | Networks advances in technology or practice flow through, and gradually populate, large scale social networks. [5] |
|  | Interventions They can do this because they possess attributes that make them attractive to different kinds of ‘adopters’. [5] |
|  | Networks are important an antecedent conditions for implementation processes, because they provide relational contexts for the reciprocal chains of interactions and flows of information that form social systems. [5] |
|  | Networks The mechanisms involved in flows of ideas and innovations spread are often unclear, but are assumed to be like those of mimesis or contagion. However they work, networks form relational pathways through which different kinds of work are done. This means that they are accomplishments rather than static structures, and that these accomplishments include information flows and practices of operationalization of the complex intervention. [5] |
|  | Networks may overlay relatively ‘open systems’ that are diffuse and unbounded, and they often transcend formal institutional boundaries. [5] |
|  | Networks might be a population dispersed over many organizations of different sizes, and distributed in social space. Or, they may overlay relatively ‘closed systems’ that appear to be highly structured and bounded. These may be specific organizations, or work groups, like those discussed by Whitten in her work on the diffusion of telemedicine services. [5] |
|  | Networks may also take the form of highly structured and bounded networks that exist within – or between – organizations. An interesting example is that of the networks involved in designing, delivering and participating in large randomized controlled clinical trials. These can be complex and widely distributed (often internationally) but remain highly structured and have robust mechanisms to ensure their closure. [5] |
|  | Field is defined as a ‘fundamental unit’ for collective action that takes the form of a ‘social order where actors (who can be individual or collective) interact with knowledge of one another under a set of common understandings about the purposes of the field, the relationships in the field (including who has power and why), and the field’s rules’. [5] |
|  | Agents work together in skilled ways to achieve goals and facilitate the engagement and co-operation of others. [5] |
|  | Social skill that proves pivotal to the construction and reproduction of local social orders (…) Social life revolves around getting collective action, and this requires that participants in that action be induced to cooperate. Sometimes coercion and sanctions are used to constrain others. But often, skilled strategic actors provide identities and cultural frames to motivate others. [5] |
|  | Participants are characterized by a variety of context-dependent affiliations, social roles, and rules in the form of social norms and conventions. These may include the capability to define and regulate conduct by consensual or coercive means. [5] |
|  | Social norms: institutionally sanctioned rules that give structure to meanings and relations within a social system, and that govern agents’ membership, behavior and rewards within it. They frame rules of membership and participation in a complex intervention. [5] |
|  | Social roles: socially patterned identities that are assumed by agents within a social system, and that frame interactions and modes of behavior. They define expectations of participants in a complex intervention. [5] |
|  | Material resources: symbolic and actual currencies, artifacts, physical systems, environments that reside within in a social system, and that are institutionally sanctioned, distributed and allocated to agents. They frame participants’ access to those material resources needed to operationalize the complex intervention. [5] |
|  | Cognitive resources: personal and interpersonal sensations and knowledge, information and evidence, real and virtual objects that reside in a social system, and that are institutionally sanctioned, distributed and allocated to agents. They frame participants’ access to knowledge and information needed to operationalize the complex intervention. [5] |
|  | Implementation of a complex intervention occurs when agents deliberately attempt to initiate its incorporation within a social system, in a way that modifies the operation of that system and changes its possible outcomes. It thus affects the social roles, norms and conventions that govern the conduct of agents, and the material and informational resources available to them, within a set of dynamic and contingent interactions. [5] |
|  | Intervention is disposed to normalization into practice if the social system in which it is located is one that provides normative and relational capacity – through which agents resource, cooperate, and coordinate their investments and contributions to its use. If capability cannot be sustained, then the embeddedness of the complex intervention will be threatened as its context of action decomposes. [5] |
|  | Agency a temporally embedded process of social engagement, informed by the past (in its habitual aspect), but also oriented toward the future (as a capacity to imagine alternative possibilities) and toward the present (as a capacity to contextualize past habits and future projects within the contingencies of the moment). [5] |
|  | Individual intentions*:* agents’ readiness to translate individual beliefs and attitudes into behaviors that are congruent, or not congruent, with system norms and roles. They frame individual motivation to participate in a complex intervention. [5] |
|  | Shared commitments*:* agents’ readiness to translate shared beliefs and attitudes into behaviors that are congruent, or not congruent, with system norms and roles. They frame shared commitment of participation in a complex intervention. [5] |
|  | Realizing agents’ capability to implement a complex intervention into action to achieve their goals depends on them being disposed to do so. These dispositions are expressed through individual attitudes and intentions, and shared values and commitments. These may depend on agents’ beliefs about attributes of the complex intervention and their beliefs and experiences of capability. [5] |
|  | Potential cannot be sustained, then the embeddedness of the complex intervention will be threatened as agents’ commitments are withdrawn. [5] |
|  | Bandura says ‘To be an agent is to intentionally make things happen by one’s actions. Agency embodies the endowments, belief systems, self-regulatory capabilities and distributed structures and functions through which personal influence is exercised, rather than residing as a discrete entity in a particular place. The core features of agency enable people to play a part in their self-development, adaptation, and self-renewal with changing times’. [5] |
|  | Bandura says ‘Forms of joint action can unite two or more individuals towards a shared end. In joint action, disparate individuals are coordinated in such a way that they become centered on each other (…) and are able to act collectively, as if they were a single entity. In certain circumstances, then, complex structures of jointly acting individual agents are able to act as collectivities.’ [5] |
|  | Collective action leads to the definition and meeting of goals, and their operation is shaped by organizing structures and social norms. [5] |
|  | Dynamic reflexivity, continuously making and acting upon their sense of the form and application of a complex intervention, at the same time appraising its effects [5] |
|  | Directed action, continuously building and acting upon the relational features, and performing the material practices needed to implement and embed the complex intervention in practice. [5] |
|  | Dynamic elements of the contexts and objects of implementation, and for the dynamic potential and actual expressions of agency. These form the social processes through which implementation is accomplished. They are not linear or sequential, but interact continuously with each other in emergent and complex ways. |
|  | Agents’ experiences of [implementation] processes vary across social time and space, as they are shaped, encouraged and confounded by other endogenous and exogenous factors. [5] |
|  | An implementation process involves agents in the intentional modification of the social systems that occupy a field, or fields, of action. [5] |
|  | Within social systems, emergent expressions of agency both shape, and are shaped by, dynamic elements of their contexts. They continuously interact to form an emergent social process. [5] |
|  | Emergent expressions of agency and dynamic elements of context continuously interact with both endogenous and exogenous contingencies and confounders. [5] |
|  | Agents work to negotiate the effects of interactions, contingencies, and confounders. They seek to make these plastic and shape them through their agentic contributions, and thus to govern the conduct of an implementation process and its outcomes. [5] |
|  | Collective action Participants in implementation contribute to their progress through work that achieves intervention coherence, cognitive participation, collective action and reflexive monitoring. [6] |
|  | Complex adaptive systems that form the dynamic environment(s) in which implementation processes are situated. [6] |
|  | Coupling is relations of dependence between actors, intervention components and dynamic elements of contexts. [6] |
|  | Elasticity The extent to which contexts can be stretched or compressed in ways that make space for intervention components and allow them to fit [[89](https://implementationscience.biomedcentral.com/articles/10.1186/s13012-016-0506-3#ref-CR89)]. [6] |
|  | Emergence is the way in which the ‘global behaviour of a system results from the actions and interactions of agents’ and unfolds unpredictably over time and across space [6]. |
|  | Normative restructuring changes the norms, rules and resources through which participation in implementation processes is structured. [6] |
|  | Plasticity is the extent to which interventions and their components are malleable and can be moulded to fit their contexts. [6] |
|  | Relational restructuring is changes to the ways that participants in implementation processes are organised and relate to each other. [6] |
|  | The contexts in which implementation processes are located can be characterised as complex adaptive social systems; [6] |
|  | The importance of intervention plasticity (that is, the extent to which users can mould them to fit a particular context) and contextual elasticity (that is, the extent to which users can mould elements of the environment to allow a set of intervention components space to work); [6] |
|  | Adaptive work explains the ways that participants’ contributions to implementation processes (resource mobilisation and collective action) lead to adaptive work (normative and relational restructuring), and so shape implementation outcomes; [6] |
|  | Implementation tends towards a quite pragmatic definition. It is constructed as *action in response to a call (or desire, or expectation, or command) for change* through which people are *asked (or want, or are expected, or are instructed) to do something new or different.* [6] |
|  | Examples of implementation are abundant in healthcare, and they can be understood as initiating and sustaining the mobilisation of structural capacity (rules and resources), and cognitive potential (readiness and commitment), in the service of collective action. [6] |
|  | Conceptualising context in terms of its relation to the individual—as in a concentric circle [6] |
|  | Context as a *place.* It can be mapped to depict spatial relations between the loci of clinical practices, organisational systems and policy problems. [6] |
|  | Context as a *process* rather than a *place* may be more useful. It acknowledges that the context in which implementation takes place is the product of continuous accomplishments that require constant work to hold together and keep moving forward. [6] |
|  | Context is a dynamic accomplishment not a fixed organisational structure or institutional entity but as unstable, unfolding, process. [6] |
|  | Context should be thought about as a set of ongoing accomplishments, rather than as concrete structures, the way we understand ‘contexts’ might change. [6] |
|  | *Adaptive* mechanisms at work in complex adaptive systems. Social systems are self-organising and self-stabilising only to the extent that human actors invest effort in making them so and only to the degree that human actors work with mechanisms that produce and sustain self-organisation. [6] |
|  | Participants’ actions shape, and are shaped by, the mechanisms at work in these systems. [6] |
|  | There are two important consequences of understanding the contexts in which implementation processes are enacted in terms of complex adaptive systems. First, it acknowledges that these processes are emergent. They unfold, over time and between settings, and are shaped by many different factors. [6] |
|  | Interactions between these factors may lead to turbulence and other unanticipated effects. [6] |
|  | We ought to consider the work of implementation not just in terms of operationalising some new technique, technology or organisational practice but also in terms of accomplishing order and predictability and damping down turbulence. [6] |
|  | Intervention components are coupled to each other and how they are coupled to dynamic elements of context. The more tightly coupled intervention components are, the less discretion in resource mobilisation and actors’ contributions are available to participants in their implementation and the less traction the intervention gains. [6] |
|  | The more loosely coupled intervention components are, the more discretion in resource mobilisation and actors’ contributions are available to participants in their implementation. [6] |
|  | To understand organisational aspects of implementation better, we need to explore the ways that they are shaped by the behaviours and actions of participants as they negotiate the normative and relational environment in which they are set. [6] |
|  | Negotiations mediate between implementation (resource mobilisation and actors’ contributions) and their outcomes (experienced workability and integration and the embeddedness of interventions). [6] |
|  | N*ormative restructuring* occurs when negotiating the implementation of intervention components in a complex adaptive system leads to modifications to the conventions, rules and resources that participants experience as providing the scaffolding for everyday behaviour and action. [6] |
|  | ‘Successful’ interventions seem to ‘restructure and reinforce new practice norms and associate them with peer and reference group behaviours’. [6] |
|  | Normative restructuring leads to changes in participant behaviour and system dynamics over time, but it also involves interactions with intervention components themselves. [6] |
|  | Intervention *plasticity*—the extent to which their users experience them as malleable and can mould them to fit their immediate contexts. The more plastic intervention components are, the more that their users have discretion about how to deploy them in practice. [6] |
|  | R*elational restructuring* occurs when negotiating the implementation of intervention components in a complex adaptive system leads to changes in the structure and conduct of the interpersonal interactions and group processes that make collective action possible. [6] |
|  | As participants enact their contributions to an implementation process, their accountabilities to each other are reworked. Here, an important property of the expression of those relations is their *elasticity*—the degree to which they can be stretched and moulded to give users room for manoeuvre as they operationalise intervention components. [6] |
|  | Discretion that stems from the experienced plasticity of intervention components and the room for manoeuvre that users find when implementation environments are characterised by elasticity are important. [6] |
|  | Intervention components are inflexible and rigidly applied, they require high levels of commitment from their users—and where this cannot be guaranteed, they require specialist practitioners or facilitators—because the turbulent flows and varying magnitude of events that are associated with complex adaptive social systems make them difficult to routinely embed in practice. [6] |
|  | Inelastic implementation environments are often characterised by rigidly formed group processes and inflexible and impermeable organisational structures. These reduce the room for manoeuvre available to participants in implementation processes and mean that the transportability of intervention components between settings is inhibited. [6] |
|  | Actors/Participants in implementation processes need to work to sustain an orderly pattern of social interactions and relations and a predictable flow of events in the face of complexity. This work is a basic underpinning of all forms of human association. So, normative and relational restructuring can be observed as continuously taking place over time and between settings. [6] |
|  | When intervention studies ‘fail’, it may be because participants have been unable to perform the degree of restructuring that is necessary to do implementation work. Restructuring is an important, but poorly understood, *adaptive* element of implementation processes. [6] |
|  | Contexts are dynamic and are subject to restructuring processes that take within complex adaptive social systems - these elements relate to each other and define a process made up of feedback loops that continuously shape and reshape implementation fidelity and outcomes. This occurs not just over time but also between settings as contextual factors affect [6] |
|  | Coupling – tight coupling of intervention components and limits on discretion about action leads to the minimisation of available space for discretion and negotiation. [6] |
|  | *Adaptive self-organisation* in complex and emergent social systems. These mechanisms are important elements of implementation processes. [6] |
|  | Interactions between the micro- and meso-level theoretical propositions on which NPT has been built and macro-level models through which we can explore the workings of ‘whole systems’ and their effects. [6] |
|  | Implementation processes as clearly defined, linear, finite projects. Scaling up and scaling out are the translational efforts that take healthcare interventions beyond the closed system of the evaluation study into ‘real world’ contexts. [6] |

**Table 2. NPT coding variables – First pass coding manual**

| **NPT construct and source references** | | **Verbatim extracts from example texts** | **Code** |
| --- | --- | --- | --- |
| **Coherence** | Coherence building that makes interventions and their components meaningful: participants contribute to enacting intervention components by working to make sense of its possibilities within their field of agency. They work to understand how intervention components are different from other practices, and they work to make them a coherent proposition for action [7]. | *coherence* was achieved around the CDSS despite local context variation. Across all three sites there was agreement that the CDSS was suitable for the (varied) tasks and that appropriate resources were in place to enable effective implementation, although these varied between settings. There were differences between settings where the CDSS replaced an established system with existing staff and where the service and/or the staff were new and the work of establishing coherence had to be altered to reflect this. It was clear that knowledge, experience and work identities built through doing call-handling work influenced the coherence of the CDSS for staff in the different settings. What is especially interesting in the wider policy context – where this same CDSS is now being used to support a national ‘111’ urgent care service (…) is that coherence was not just a local ‘problem’, it was necessarily underpinned by wider understandings and discourses for example about the necessity of rationing and the need to modify caller/patient behaviour and beyond that the very legitimacy of evidence based medicine and the kinds of expert knowledge which underpinned the CDSS [8]. | COHE |
|  | **Differentiation**: An important element of sense-making work is to understand how interventions and their components, and prior practice are different from each other. [9] | In order to invest in ERAS individuals needed to be able to differentiate its practices favourably with those enacted pre-implementation. This required *coherence* work in understanding the potential patient benefits allied to its introduction. Participants provided divergent accounts when they compared ERAS to previous practice. A number of participants asserted that the introduction of ERAS had brought about considerable changes to their day-to-day practice. These changes included positive adjustments in the management of patients and required patients to play a more active role in their own recovery [10]. | CODI |
|  | **Communal specification**: Sense-making relies on people working together to build a shared understanding of the aims, objectives, and expected benefits of interventions and their components. [9] | Another barrier to coherence was lack of communal specification, since not everyone considered they had been informed about the study or understood its aims and processes. This caused implementation problems for the homes and the research team. For the homes, the researchers’ reasons for examining potential benefits from the intervention to have a positive impact on the culture of care had not been strongly reflected [11]. | COCS |
|  | **Individual specification**: Sense-making has an individual component, too. Here participants in coherence work need to do things that will help them understand their specific tasks and responsibilities around interventions and their components. [9] | One respondent felt discussing the new way to view the patients with the staff was a delicate issue. In the old care model, patients were usually only informed about the treatment whilst now, in the care model, patients were to be seen as partners. This was regarded as a shift in power and, at least for some physicians, it would be difficult to get used to.  Respondent 8: “It’s quite a delicate issue to talk about the physicians not giving patients total participation in the care. So, there is a lot of work to do in that perspective. It may have something to do with tradition, the physicians know best, and now we are changing the focus to the patient, that the patient knows best” [12]. | COIS |
|  | **Internalization**: Sense-making involves people in work that is about understanding the value, benefits and importance of interventions and their components. [9] | At this stage (initial introductory meetings), the value of the intervention was purely based on individuals’ interpretation of the information given by the research team and the “fit” with their own interests. The GPs in General Practice 8 provided their views at the end of the introductory meeting, saying that they liked the structure and more systematic approach to caring for people with OA and concluded that “it is nice to be able to try something that may make a difference” [13]. | COIN |
| **Cognitive Participation** | Cognitive participation that forms commitment around an intervention and its components: participants contribute to enacting intervention components through work that establishes its legitimacy and that enrols themselves and others into an implementation process. This work frames how participants become members of a specific community of practice [7] | Cognitive participation relates to the work that participants undertake to build up and sustain a community of practice around an intervention. In terms of CST, participants identified training as an important factor in generating their own and their colleagues’ interest in CST and thus ensuring all stakeholders were involved. Staff were further motivated to continue running the groups within their service through observing the direct beneficial effects of CST on clients [14]. | COGP |
|  | **Initiation**: A core problem is whether or not key participants are working to drive interventions and their components. forward. [9] | Participants described the new SDM work as requiring leaders to define the work, and then enrolling others to contribute collectively to the process. Identifying leadership support for SDM was challenging: clinical teams are not simple hierarchical units, and substantial autonomy exists, especially for experienced clinicians [15]. | CPIN |
|  | **Enrolment**: Participants may need to organize or reorganize themselves and others in order to collectively contribute to the work involved in interventions and their components. [9] | Clinic participants also re- ported that the intervention provided a model for improved interprofessional team collaboration, resulting in a greater understanding of clinicians’ roles and skill sets. Huddles were viewed as worth creating and maintaining, both for interprofessional team and patient benefits. Participants identified that the majority of patients were satisfied with the HT interprofessional approach to primary care [16] | CPEN |
|  | **Legitimation**: An important component of relational work around interventions and their components is the work of ensuring that other participants believe it is right for them to be involved, and that they can make a valid contribution to it*.* [9] | The respondents offered several explanations for resistance or lack of engagement: some staff felt that health promotion activities overstretched users’ resources and thus had a negative impact on their quality of life; others argued that health promotion activities did not respect personal preferences of users and staff (…) One of the important implementation ideas in the SLIPS was the concept of staff being role models for health promotion. As role models staff was expected to participate in different health promotion activities (like joining users for walks and meals) and to display a healthy lifestyle at work. In the four providers, such expectations were formulated and formalised by management or by key implementation staff to different extents. However, in all cases some staff did not buy into this idea; they felt that the elements of smoking cessation and healthier meals interfered with their usual lifestyle and personal preferences [17] | CPLE |
|  | **Activation**: Once it is underway, participants need to collectively define and enact the actions and procedures needed to sustain interventions and their components and to stay involved. [9] | While, overall, this system has worked well, many participants referenced instances of long wait times and rerouting of calls to reach the neonatologist. Based on the care teams' appraisal and experience with this process, they suggested modeling the teleneonatology service activation after the emergency department's response system, for immediate and direct connection. Other suggestions include making the technology simple enough for ease of use, and to mount a camera (which can be controlled by the remote neonatologist) to the baby warmer [18]. | CPAC |
| **Collective Action** | Collective action through which effort is invested in an intervention and its components: participants mobilize skills and resources and make a complex intervention workable. This work frames how participants realize and perform intervention components in practice [7]. | The daily tasks involved in carrying out POC testing were deciding which tests (if any) to take for each patient when they arrived; communicating this to others; taking the blood; running the tests; examining the results; communicating the results to others; and deciding what action to take accordingly. This work was allocated to different staff according to their skills and availability. Close teamwork appeared key to ensuring that each task was performed by an appropriate person at the necessary time [19]. | CACT |
|  | **Interactional Workability**: This refers to the interactional work that people do with each other, and with the components of interventions and their components when they seek to operationalize them in everyday settings. [1] | The rural allied health team indicated that telehealth technology provided ‘a whole range of other capabilities’, and considered it ‘safe and it’s appropriate and it’s an equivalent, if not better, sort of service that you can provide’. They were committed to the notion that telehealth could balance the unequal access to services across geographical locations, and were keen to pursue innovative ways of using telehealth technologies to allow them to provide complex distant therapy. In contrast to rural and experienced telehealth clinicians who were keen to utilise technology as part of their role and to deal with distance and isolation, urban clinicians with no exposure to telehealth reported more reservations about the safety and suitability of providing rehabilitation through telehealth. They generally felt that telehealth should be reserved for ‘people who are more autonomous and more capable and … straightforward’, rather than ‘real’ rehabilitation patients with complex issues. They felt that people who required rehabilitation often require a ‘hands on’ approach [20] | CAIW |
|  | **Relational Integration**: This refers to the knowledge work that people do to build accountability and maintain confidence in interventions and their components and in each other as they use them. [1] | Enhanced collegial discussion about FV and adherence to the safety measures, such as the home visiting policy and procedures introduced in the MOVE model, were important for nurses to feel safe and undertake the FV work. As implementation progressed, intervention nurses felt safer than comparison nurses when attending home visits (S2-IG 82.1%, CG 62.3% (*p* = 0.02)). Relationships within teams and with FV services varied across the MCH intervention teams. High workloads, time constraints and a lack of nursing staff or relievers in some centres impacted on the organisation of the FV work at times. The nurse mentor role to provide secondary consultation, linkage to FV services and support for other MCH nurses had varied success. Due to time constraints and the often solo nature of MCH practice, most nurses preferred to discuss clinical issues with a nurse friend or co-worker at the time rather than try to contact the designated MOVE nurse mentor, with only 38% of nurses using the nurse mentor role early in the trial. This increased to 52% as time went on. If the nurse was not comfortable speaking and had insufficient time or access to the nurse mentor, then this aspect of the model was lost [21]. | CARI |
|  | **Skill set Workability**: This refers to the allocation and training work that underpins that is built up around interventions and their components as it is operationalised in the real world. [1] | A key theme identified in the literature and through this study is the need for more training for practitioners. This includes training both in professional education and continuing educational opportunities for all practitioners. Medical, nursing and allied health education programs need to improve LGBT curriculum content [[2](https://journals.plos.org/plosone/article?id=10.1371/journal.pone.0215873#pone.0215873.ref002), [64](https://journals.plos.org/plosone/article?id=10.1371/journal.pone.0215873#pone.0215873.ref064)–[66](https://journals.plos.org/plosone/article?id=10.1371/journal.pone.0215873#pone.0215873.ref066)]. Providing education on general terminology, healthcare needs specific to the transgender population, and practitioners’ role in providing healthcare for this population will better prepare new practitioners for serving this community. Increased access to continuing education with LGBT content will help to increase the knowledge and skill of current practitioners. Embedding LGBT content within current programs of continuing education may increase awareness more than having specific LGBT courses (…). Embedding it in current programs may bring awareness to the concepts and highlight the need for practitioners to seek out more specific training to address their learning gaps [22] | CASW |
|  | **Contextual Integration**: This refers to the resource work – supporting interventions and their components through the allocation of different kinds of resources and the execution of protocols, policies and procedures. [1] | Since POs were able to self-select into the pilot, the alignment of PO priorities with participation in a pilot on care management was a good fit. The leadership in all POs voiced interest in providing care management to patients within their PO as a means of improving patient outcomes, easing burden on providers of handling complex patients, and to meet health care standards and reimbursement policies such as patient centered medical home recognition, accountable care, and meaningful use. Therefore, in this study overall organizational support was not found to be variant. Where organizational support emerged as an issue related more to resources and support for the care management program relative to the needs and goals of the program. The most common issue here was not having either enough care managers or enough care manager protected time to do care management for the number of patients needing it. So in well-normalized programs, there was a sense of “rationing” of the care manager. Because the program was being used so much more and there was a capacity constraint at the practice level with the practice-based care manager structure, the practices in these POs voiced more concern about lack of care manager capacity (POs C_P_and E_P_). Lack of resources was evident in other ways such as lack of space for patient visits or access to phone lines to make longer calls [23]. | CACI |
| **Reflexive Monitoring** | Reflexive monitoring through which the effects of an intervention and its components are appraised: participants contribute to enacting intervention components through work that assembles and appraises information about their effects and utilize that knowledge to reconfigure social relations and action [7]. | Data provision by the laboratories proved to be difficult despite the standardized format. The database manager at the central level reported he had to put much effort in getting the data from the system administrator from the laboratories because they did not prioritize data delivery. It was reported by them that saving the data extraction queries, as the research group suggested, for use in the next time period was increasingly helpful in the course of the implementation period. By fine‐tuning these queries after each extraction, the quality of the delivered data improved [24]. | REMO |
|  | **Systematization**: participants in interventions and their components may seek to determine how effective and useful it is for them and for others, and this involves the work of collecting structured information in a variety of ways. [9] | Feedback was never provided to staff on the effect of the AKI e-alert *“I haven’t had any feedback since the new version (of the AKI e-alert) went in actually(...) I don’t know whether there is a formal mechanism for that getting to anyone”.* (Pharmacist interview, Trust 1) [25]. | RMSY |
|  | **Communal appraisal**: participants work together - sometimes in formal collaboratives, sometimes in informal groups to evaluate the worth of interventions and their components They may use many different means to do this drawing on a variety of experiential and systematized information. [9] | The e-alert was rarely (if ever) discussed among clinicians, but participants often stated they felt that others would find it worthwhile. “*The e-alert was rarely (if ever) discussed among clinicians, but participants often stated they felt that others would find it worthwhile. “Most people I'm sure would know it's a good idea having them. That's what I'd say to someone about these alerts”*  [25]. | RMCA |
|  | **Individual appraisal**:  Participants in interventions and their components also work experientially as individuals to appraise its effects on them and the contexts in which they are set. From this work stem actions through which individuals express their personal relationships to new technologies or complex interventions. [9] | A key barrier which has not previously been identified concerned the ability of case managers to identify, and act on, emerging patient and carer needs; we identified examples of missed and unmet needs for all three case managers. One case manager explicitly attributed this to the timing of the intervention; a study of case management for people with early symptoms of dementia and their carers similarly found that case managers did not feel the intervention was needed at this point [26]. | RMIA |
|  | **Reconfiguration**: appraisal work by individuals or groups may lead to attempts to redefine procedures or modify interventions and their components[9] | Aligning IPC guidelines with local clinical context is an essential means to reduce the sense of *dissonance* and represents a critical step forward towards successful implementation. Some strategies described in the literature to promote alignment include: integration of IPC recommendations within other established programmes; and education and audit interventions acknowledging the positive and negative beliefs of staff on IPC practices [27]. | RMRE |
| **Contextual features of interventions and their components** | Contexts provide social structural and social cognitive resources, and we can frame these in relation to different mechanisms. [These] are concerned with any context’s capacity to accommodate implementation processes [7]. |  | CNMP |
|  | **Plasticity**: The extent to which interventions and their components are malleable and can be moulded to fit their contexts. [28] [6] | There was also concern, commonly expressed in evaluations of cascade models, about the dilution of the quality of the training over time and about the degree to which it could be modified to match the needs of the particular group being trained. STORM is a relatively plastic intervention in that its format can be modified to suit the context—however some trainers seemed unaware of or uncertain about the extent to which this was allowable [29]. | CNPL |
|  | **Elasticity**: The extent to which contexts can be stretched or compressed in ways that make space for interventions and their components and allow them to fit. [6] | Initial implementation was followed in all homes by a period where the cooks adjusted the menus or recipes in the light of feedback from clients and/or care staff. This process highlighted the limitations of the training. While some cooks simply reinstated popular dishes from the baseline menus, others tried to follow the principles underlying the modified menus. However, they found it difficult to manage the tension between meeting the nutrition guidelines and client preferences [30] | CNEL |
|  | **Coupling**: Relations of interdependence between people, interventions and their components, and the contexts in which they are working. [6, 31] | Generally, then, variability in how delirium was understood among different groups of staff and the lack of investment at organisational level in respect of training and education meant that delirium identification had low coherence, in NPT terms. Delirium diagnosis was primarily effected through use of observational cues – although how these were interpreted and acted upon depended on the expertise of those making the observations. Thus, management practices following on from observations reflected the skills and interests of individual professionals rather than collective staff and ward response [32] | CNCU |
| **Negotiated outcomes** | NPT characterizes contexts as dynamic; an important prediction of the theory is that how participants in implementation processes relate to other actors, processes and structures to be found in their environments really does matter [7]. | The external and internal partnership building were key and also strategic, so as not to impose ERAS but to co-create it from the ground up. This relational work, as framed in the NPT, is deceptively complex as it involves convincing others that this is a legitimate improvement programme worth participating in without devaluing their current practice and beliefs. The interprofessional and interdepartmental relationships the champion teams established appeared to lay an important foundation for accepting changes and the data reports as meaningful and embedding ERAS into everyday practice [33]. | NEOT |
|  | **Norms and Normative restructuring:** Changes to professional norms, rules and resources as a result of working with interventions and their components [6] | The first theme, trusting and embedding new relationships, is a reminder that while locally-led innovation is designed to address local problems, convincing others of its value is core work. This is particularly so when the innovation challenges professional norms and involves changes to traditional delivery models and renegotiation of professional roles (…). In this case, the findings are consistent with previous research which has indicated that the success of such innovations is dependent on the trust of all involved and the credibility of clinicians (…) [34]. | NNRE |
|  | **Roles and Relational restructuring:** Changes to the ways that people are organised and relate to each other as a result of working with interventions and their components [6] | The CMs became “everyday representatives” for the secondary sector and were responsible for acting as bridge‐builders between hospital psychiatry and general practice. Previous research on Nurse Practitioners/Advanced Nurse Practitioners in general practice (…) has shown that if the clinics are not involved at an early stage and prepared thoroughly for the Nurse Practitioner's arrival, their integration in general practice is hampered. Preparation involves practical issues, a clearly defined role for the nurse practitioner, and *organizational leadership*, meaning that the managers of the responsible organization must be involved in the process of defining and supporting the role (…) The challenges also pointed towards a lack of managerial co‐ordination of, and responsibility for, the practical issues associated with the CM's role in general practice. (…) This meant that on many occasions, the CMs had to take on the role of implementation ambassadors assuming responsibility for maintenance of the collaborative care model [35]. | NNRR |
|  |  | The bed-monitoring technologies were felt to be useful in helping staff identify patterns in resident behaviour and explore reasons for these behaviours. The bed sensors at Sycamore Lane were capable of recording clinical data such as heart rate, but the manager reported that *“it’s not something that we use readily”*, and this functionality was never observed in use during the present study. The location-based system at Conifer Gardens was similarly able to record data, including information about resident mobility activity. This functionality had initially been anticipated as potentially useful for enhancing clinical understanding, however, the Occupational Therapist reflected that the time needed to analyse and interpret these data had been *“a job in itself”* and thus has been difficult to integrate into daily practice. There were questions about the clinical utility of some of the data, which appeared to become more pronounced when considering the financial expense of the technology [36] | NNIN |
|  | **Intervention success:** experienced workability and integration in contexts [28] | Participants identified that the majority of patients were satisfied with the HT interprofessional approach to primary care. Overall, they deemed the intervention worthwhile and sustainable. Changes to interprofessional teamwork structure and processes were considered possible to sustain, suggesting that normalization had occurred [16]. | NNIS |
| **Potential** | [These are] concerned with the ways in which participants relate to the activities involved in implementing intervention components. These are social cognitive resources [7]. | At the beginning of the study potential was positive for the intervention across all sites (…) co-ordinators were provided with office accommodation and administrative assistance; staff from local agencies were released for training as SFP 10–14 facilitators. Some practitioners were strongly motivated to provide direct support (e.g. working as a facilitator) and indirect support (e.g. referring families to the programme) [37]. | POTL |
|  | **Individual readiness:** Participants’ readiness to translate individual beliefs and attitudes about interventions and their components into behaviours that are congruent, or not congruent, with (new) system norms and roles. [5] | Participants in this study were mainly enthusiastic and committed to enacting the intervention. They agreed that the fracture prevention service was necessary, and they were highly supportive of the service in action. Those that were not tended to be characterized by their peers as negative or unsupportive personalities [38]. | POIR |
|  | **Shared commitments:** Participants’ readiness to translate shared beliefs and attitudes about interventions and their components into behaviours that are congruent, or not congruent, with (new) system norms and roles. [5] | …it was clear that this lack of support—sometimes manifest in open obstruction from a small number of clinicians—was a problem that reflected other longstanding complexities in relations between different specialists and within hospital departments [38].. | POSC |
|  | **External (exogenous) processes and events** that shape the capacity of participants to implement and operationalize interventions and their components (e.g. Austerity, Covid) [5] | Alongside financial resources (e.g. for employment of a coordinator) another challenge identified in most counties was the need to maintain and coordinate a network of facilitators from multi-agency partnerships who could staff programmes. There was uncertainty as to whether agencies would continue to provide time off in lieu to staff who worked as facilitators on the programme outside of normal working hours [37]. | POEX |
|  | **Internal (endogenous) processes and events** that shape the capacity of participants to implement and operationalize interventions and their components (e.g. staff shortages, strategic initiatives) [5] | In addition, it indicates the importance of *designing intervention objects that are resilient to organisational turbulence*. Many healthcare interventions take place in complex settings wherein emergent and contextual factors can modify the conditions of implementation. Providing organisational ‘closure’ through monitoring and support of a key person is important in mitigating these effects; however, objects of interventions (eg, procedures, training seminars, technologies, etc) are important in this process [39]. | POEN |
|  | **Intervention Timing** |  | TIIN |
|  | **Time** as a corporate resource | "… Another factor that detracted from routine use was lack of resources such as not enough time allotment for care management work, the care manager being pulled to complete other tasks and lack of other material needs such as space and time to complete the care management’[23] | TICR |
|  | **Time** as duration, | "Nonetheless over time, checklist use had improved as senior nurses persisted with the implementation of *PTB*, despite disparaging remarks made by some anaesthetists."[40] | TIDU |
|  | **Time** as a source of variation | Staff have to find subversive ways of communicating beyond an increasing focus on roles. In practice two, the action for the practice to take forward following the problem-solving session was to institute a mandatory coffee break [41]. | TIVA |
|  | **Time** as a constraint on personal agency | "Lack of time was also a factor that influenced GPs use of Check Up GP. Using the app inherently added additional time to a consultation and GPs felt they had to rush to address all issues raised. For one GP who always ran to schedule, there was often not enough time for patients who did not arrive early to complete Check Up GP in the waiting room as this GP was not prepared to run even a little over time or to wait for young people to complete Check Up GP. Another GP felt that it was feasible to continue to use Check Up GP as part of young people’s routine care, though not at certain times, such as on weekends when only one GP works or during very busy periods [42] | TIAG |

REFERENCES

1. May C: **A rational model for assessing and evaluating complex interventions in health care**. *BMC Health Services Research* 2006, **6**(86 ):1-11.

2. May C, Finch T, Mair F, Ballini L, Dowrick C, Eccles M, Gask L, MacFarlane A, Murray E, Rapley T: **Understanding the implementation of complex interventions in health care: the normalization process model**. *Bmc Health Serv Res* 2007, **7**(1):148.

3. May C, Finch T: **Implementing, embedding, and integrating practices: an outline of normalization process theory**. *Sociology* 2009, **43**(3):535-554.

4. May C, Mair FS, Finch T, MacFarlane A, Dowrick C, Treweek S, Rapley T, Ballini L, Ong BN, Rogers A *et al*: **Development of a theory of implementation and integration: Normalization Process Theory**. *Implementation Science* 2009, **4**(29).

5. May C: **Towards a general theory of implementation**. *Implementation Science* 2013, **8**(1):18.

6. May CR, Johnson M, Finch T: **Implementation, context and complexity**. *Implementation Science* 2016, **11**(1):141.

7. May C, Rapley T, Finch T: **Normalization Process Theory**. In: *International Handbook of Implementation Science.* Edited by Nilsen P, Birken S. London: Edward Elgar; 2020: 144-167.

8. Pope C, Halford S, Turnbull J, Prichard J, Calestani M, May C: **Using computer decision support systems in NHS emergency and urgent care: ethnographic study using normalisation process theory**. *Bmc Health Serv Res* 2013, **13**.

9. May C, Finch T: **Implementation, embedding, and integration: an outline of Normalization Process Theory**. *Sociology* 2009, **43**(3):535-554.

10. Sutton E, Herbert G, Burden S, Lewis S, Thomas S, Ness A, Atkinson C: **Using the normalization process theory to qualitatively explore sense-making in implementation of the enhanced recovery after surgery programme: "it’s not rocket science"**. *PLoS ONE* 2018, **13**(4).

11. Keenan J, Poland F, Manthorpe J, Hart C, Moniz-Cook E: **Implementing e-learning and e-tools for care home staff supporting residents with dementia and challenging behaviour: A process evaluation of the ResCare study using normalisation process theory**. *Dementia* 2018.

12. Alharbi TS, Carlström E, Ekman I, Olsson L-E: **Implementation of person-centred care: management perspective**. *Journal of Hospital Administration* 2014, **3**(3):p107.

13. Morden A, Brooks L, Jinks C, Porcheret M, Ong BN, Dziedzic K: **Research “push”, long term-change, and general practice**. *Journal of health organization and management* 2015, **29**(7):798-821.

14. Dickinson C, Gibson G, Gotts Z, Stobbart L, Robinson L: **Cognitive stimulation therapy in dementia care: exploring the views and experiences of service providers on the barriers and facilitators to implementation in practice using Normalization Process Theory**. *International Psychogeriatrics* 2017, **29**(11):1869-1878.

15. Lloyd A, Joseph-Williams N, Edwards A, Rix A, Elwyn G: **Patchy 'coherence': using normalization process theory to evaluate a multi-faceted shared decision making implementation program (MAGIC)**. *Implement Sci* 2013, **8**:102.

16. Valaitis R, Cleghorn L, Dolovich L, Agarwal G, Gaber J, Mangin D, Oliver D, Parascandalo F, Ploeg J, Risdon C: **Examining Interprofessional team structures and processes in the implementation of a primary care intervention (Health TAPESTRY) for older adults using normalization process theory**. *BMC Family Practice* 2020, **21**.

17. Burau V, Carstensen K, Fredens M, Kousgaard MB: **Exploring drivers and challenges in implementation of health promotion in community mental health services: A qualitative multi-site case study using Normalization Process Theory**. *BMC Health Services Research* 2018, **18**(1).

18. Asiedu GB, Fang JL, Harris AM, Colby CE, Carroll K: **Health Care Professionals' Perspectives on Teleneonatology Through the Lens of Normalization Process Theory**. *Health Sci Rep* 2019, **2**(2):e111.

19. Glogowska M, Simmonds R, McLachlan S, Cramer H, Sanders T, Johnson R, Kadam UT, Lasserson DS, Purdy S: **"Sometimes we can't fix things": a qualitative study of health care professionals' perceptions of end of life care for patients with heart failure**. *Bmc Palliative Care* 2016, **15**.

20. Shulver W, Killington M, Crotty M: **‘Massive potential’or ‘safety risk’? Health worker views on telehealth in the care of older people and implications for successful normalization**. *BMC Medical Informatics and Decision Making* 2016, **16**(1):131.

21. Hooker L, Small R, Humphreys C, Hegarty K, Taft A: **Applying normalization process theory to understand implementation of a family violence screening and care model in maternal and child health nursing practice: a mixed method process evaluation of a randomised controlled trial**. *Implement Sci* 2015, **10**(1):39.

22. Ziegler E, Valaitis R, Yost J, Carter N, Risdon C: **“Primary care is primary care”: Use of Normalization Process Theory to explore the implementation of primary care services for transgender individuals in Ontario.** *PloS one* 2019, **14**(4):e0215873.

23. Holtrop JS, Potworowski G, Fitzpatrick L, Kowalk A, Green LA: **Effect of care management program structure on implementation: a normalization process theory analysis**. *BMC Health Services Research* 2016, **16**(1):386.

24. Trietsch J, van Steenkiste B, Hobma S, Frericks A, Grol R, Metsemakers J, van der Weijden T: **The challenge of transferring an implementation strategy from academia to the field: a process evaluation of local quality improvement collaboratives in Dutch primary care using the normalization process theory**. *Journal of evaluation in clinical practice* 2014, **20**(6):1162-1171.

25. Scott J, Finch T, Bevan M, Maniatopoulos G, Gibbins C, Yates B, Kilimangalam N, Sheerin N, Kanagasundaram NS: **Acute kidney injury electronic alerts: mixed methods Normalisation Process Theory evaluation of their implementation into secondary care in England**. *BMJ Open* 2019, **9**(12):e032925.

26. Bamford C, Poole M, Brittain K, Chew-Graham C, Fox C, Iliffe S, Manthorpe J, Robinson L, team C: **Understanding the challenges to implementing case management for people with dementia in primary care in England: a qualitative study using Normalization Process Theory**. *BMC Health Serv Res* 2014, **14**(1):549.

27. Agreli H, Barry F, Burton A, Creedon S, Drennan J, Gould D, May CR, Smiddy MP, Murphy M, Murphy S *et al*: **Ethnographic study using Normalization Process Theory to understand the implementation process of infection prevention and control guidelines in Ireland**. *BMJ Open* 2019, **9**(8):e029514.

28. May C: **Agency and implementation: Understanding the embedding of healthcare innovations in practice**. *Social Science & Medicine* 2013, **78**(0):26-33.

29. Gask L, Coupe N, Green G: **An evaluation of the implementation of cascade training for suicide prevention during the 'Choose Life' initiative in Scotland - Utilizing Normalization Process Theory**. *BMC Health Services Research* 2019, **19**(1).

30. Bamford C, Heaven B, May C, Moynihan P: **Implementing nutrition guidelines for older people in residential care homes: a qualitative study using normalization process theory**. *Implement Sci* 2012, **7**(1):106.

31. Perrow C: **Normal accidents : living with high-risk technologies**. New York: Basic Books; 1984.

32. Godfrey M, Smith J, Green J, Cheater F, Inouye SK, Young JB: **Developing and implementing an integrated delirium prevention system of care: a theory driven, participatory research study**. *BMC Health Serv Res* 2013, **13**(1):341.

33. Conn LG, McKenzie M, Pearsall EA, McLeod RS: **Successful implementation of an enhanced recovery after surgery programme for elective colorectal surgery: a process evaluation of champions’ experiences**. *Implementation Science* 2015, **10**(1):99.

34. Foster M, Burridge L, Donald M, Zhang J, Jackson C: **The work of local healthcare innovation: A qualitative study of GP-led integrated diabetes care in primary health care Organization, structure and delivery of healthcare**. *BMC Health Services Research* 2016, **16**(1).

35. Overbeck G, Kousgaard MB, Davidsen AS: **The work and challenges of care managers in the implementation of collaborative care: a qualitative study**. *Journal of Psychiatric and Mental Health Nursing* 2018, **25**(3):167-175.

36. Hall A, Wilson CB, Stanmore E, Todd C: **Implementing monitoring technologies in care homes for people with dementia: A qualitative exploration using Normalization Process Theory**. *International Journal of Nursing Studies* 2017, **72**:60-70.

37. Segrott J, Murphy S, Rothwell H, Scourfield J, Foxcroft D, Gillespie D, Holliday J, Hood K, Hurlow C, Morgan-Trimmer S *et al*: **An application of Extended Normalisation Process Theory in a randomised controlled trial of a complex social intervention: Process evaluation of the Strengthening Families Programme (10–14) in Wales, UK**. *SSM - Population Health* 2017, **3**:255-265.

38. Andrews R, Boyne GA, Law J, Walker RM: **Myths, measures and modernisation: A comparison of local authority performance in England and Wales**. *Local Gov Stud* 2003, **29**(4):54-75.

39. Bracher M, Steward K, Wallis K, May CR, Aburrow A, Murphy J: **Implementing professional behaviour change in teams under pressure: Results from phase one of a prospective process evaluation (the Implementing Nutrition Screening in Community Care for Older People (INSCCOPe) project)**. *BMJ Open* 2019, **9**(8).

40. Gillespie BM, Harbeck E, Lavin J, Gardiner T, Withers TK, Marshall AP: **Using normalisation process theory to evaluate the implementation of a complex intervention to embed the surgical safety checklist**. *BMC Health Serv Res* 2018, **18**(1):170.

41. Kennedy A, Chew-Graham C, Blakeman T, Bowen A, Gardner C, Protheroe J, Rogers A, Gask L: **Delivering the WISE (Whole Systems Informing Self-Management Engagement) training package in primary care: learning from formative evaluation**. *Implementation Science* 2010, **5**(1):7.

42. Webb MJ, Wadley G, Sanci LA: **Experiences of general practitioners and practice support staff using a health and lifestyle screening app in primary health care: Implementation case study**. *Journal of Medical Internet Research* 2018, **20**(4).
